# Mitophagy in skeletal muscle: Impact of ageing, exercise and disuse

**DOI:** 10.1113/EP093041

**Published:** 2025-11-19

**Authors:** Anastasiya Kuznyetsova, David A. Hood

**Affiliations:** ^1^ Muscle Health Research Centre, School of Kinesiology and Health Science York University Toronto Canada

**Keywords:** adaptation, lysosomes, mitophagy, Parkin, skeletal muscle, transcription factor EB

## Abstract

Skeletal muscle plays an important role in whole‐body health, quality of life and regulation of metabolism. The maintenance of a healthy mitochondrial pool is imperative for the preservation of skeletal muscle quality and is mediated through mitochondrial quality control consisting of mitochondrial turnover mediated by a balance between organelle synthesis and degradation. The selective tagging and removal of dysfunctional mitochondria is essential for maintaining mitochondrial quality control and is termed mitophagy. The mechanisms of the initial stages of mitophagy involving the recognition and tagging of mitochondria within skeletal muscle are well established, but our understanding of the terminal step involving organelle degradation mediated via lysosomes is in its infancy. An assessment of the proteolytic functions to facilitate the removal and breakdown of dysfunctional mitochondria is crucial for our understanding of the mechanisms of mitophagy, which is essential for maintaining skeletal muscle health. The aim of this review is to address the current knowledge surrounding mitophagy and lysosomal function, alongside distinct physiological conditions, such as ageing, exercise and disuse, that have varying effects on mitophagy and lysosomal adaptations within skeletal muscle.

## Abstract (highlights)

- **What is the topic of this review?**
  Various physiological conditions, such as ageing, exercise and disuse, have differing effects on mitophagy, the process of removing dysfunctional mitochondria. The terminal step of this process is mediated by lysosomes, and this review highlights the importance of the interaction between mitophagy and lysosomal function in skeletal muscle.
- **What advances does it highlight?**
  This review delves into the current literature regarding the drive for various mitophagy pathways within skeletal muscle and highlights the importance of assessing and evaluating lysosomal activity as a measure of functional mitophagy. These pathways and methods are particularly important for addressing the effects of exercise, ageing and disuse within muscle.

## 1 SKELETAL MUSCLE AND MITOCHONDRIA

Skeletal muscle is the most abundant tissue in the human body, and it possesses a remarkable feature of adaptability with respect to contractile and metabolic properties. The continuous synthesis of ATP is required to facilitate muscle contraction, involving constant adjustments in the rate of energy production based on physiological conditions (Hargreaves et al., 2020). The mitochondrion is the organelle capable of ATP production largely responsible for the plastic metabolic qualities that skeletal muscle encompasses, and it must adapt to meet these diverse energetic demands (Hood et al., 2001).

The structure of skeletal muscle consists of long, multinucleated myofibres. Myofibrils composed of contractile proteins run through the length of a myofibre and contain visible sarcomeres, which are organizational units of contractile function (Frontera et al., 2015). Mitochondria are dispersed within skeletal muscle in divergent geographical areas to disburse ATP to different structural compartments of the myofibre (Hood et al., 2019). Subsarcolemmal (SS) mitochondria, located beneath the sarcolemma, possess a regular oval‐shaped phenotype, and they contribute to the energetic requirements for membrane and nuclear functions. Intermyofibrillar (IMF) mitochondria are located between myofibrils, are elongated, form a reticulated network and provide the energy for contractile functions, such as actin–myosin interactions (Cogswell et al., 1993; Vincent et al., 2019).

Mitochondria compose a relatively small proportion of cell volume in skeletal muscle (∼2%–8%) in comparison to other tissue types, such as cardiac muscle (∼30%), and this varies based on fibre type and training status (Ventura‐Clapier et al., 2019). Muscle fibre types consist of slow‐twitch and fast‐twitch fibres classified as type I and type II, respectively. Type I fibres contract slowly and are more aerobic owing to a higher mitochondrial content resulting in reduced fatiguability. Type II fibres can be classified as IIA or IIX in human skeletal muscle, and they contract more rapidly, produce more force and fatigue faster relative to type I fibres as a result of having a lower mitochondrial content (Herbison et al., 1982). Subsarcolemmal mitochondria occupy ∼15% of the total mitochondrial pool, whereas intermyofibrillar mitochondria occupy the remainder, ∼85%. The low mitochondrial content in skeletal muscle suggests that there is room for adaptations imposed by various stressors, such as exercise. In particular, the subsarcolemmal pool responds particularly well, and these mitochondria are more adaptable to alterations in energy demand than the intermyofibrillar mitochondria (Hood et al., 2001). In contrast to the positive adaptations brought about by exercise, mitochondrial content can also diminish in response to disuse or ageing. These alterations in mitochondrial content are regulated by a series of pathways that maintain, augment and control mitochondrial quality, termed mitochondrial quality control (MQC).

The MQC consists of several signalling pathways and processes to maintain skeletal muscle and mitochondrial function, thus preserving adaptive capabilities (Liu et al., 2023). Mitochondrial biogenesis involves the transcriptional and post‐transcriptional upregulation of proteins encoded by both nuclear and mitochondrial DNA, synthesizing new mitochondria. This process is important to activate in response to increased energy demands, ensuring that a sufficient mitochondrial network exists within the cell. Mitochondrial fusion and fission are processes of MQC whereby mitochondria either fuse with or segregate from one another. Furthermore, MQC also consists of a mitochondrion‐specific autophagy pathway to clear dysfunctional mitochondria, termed mitophagy (Hood et al., 2019; Liu et al., 2023). The aim of this review is to delve into processes of mitophagy, the emerging role of lysosomes and the adaptations associated with various physiological conditions, such as ageing, physical exercise and disuse.

## 2 MITOPHAGY

**FIGURE 1:**
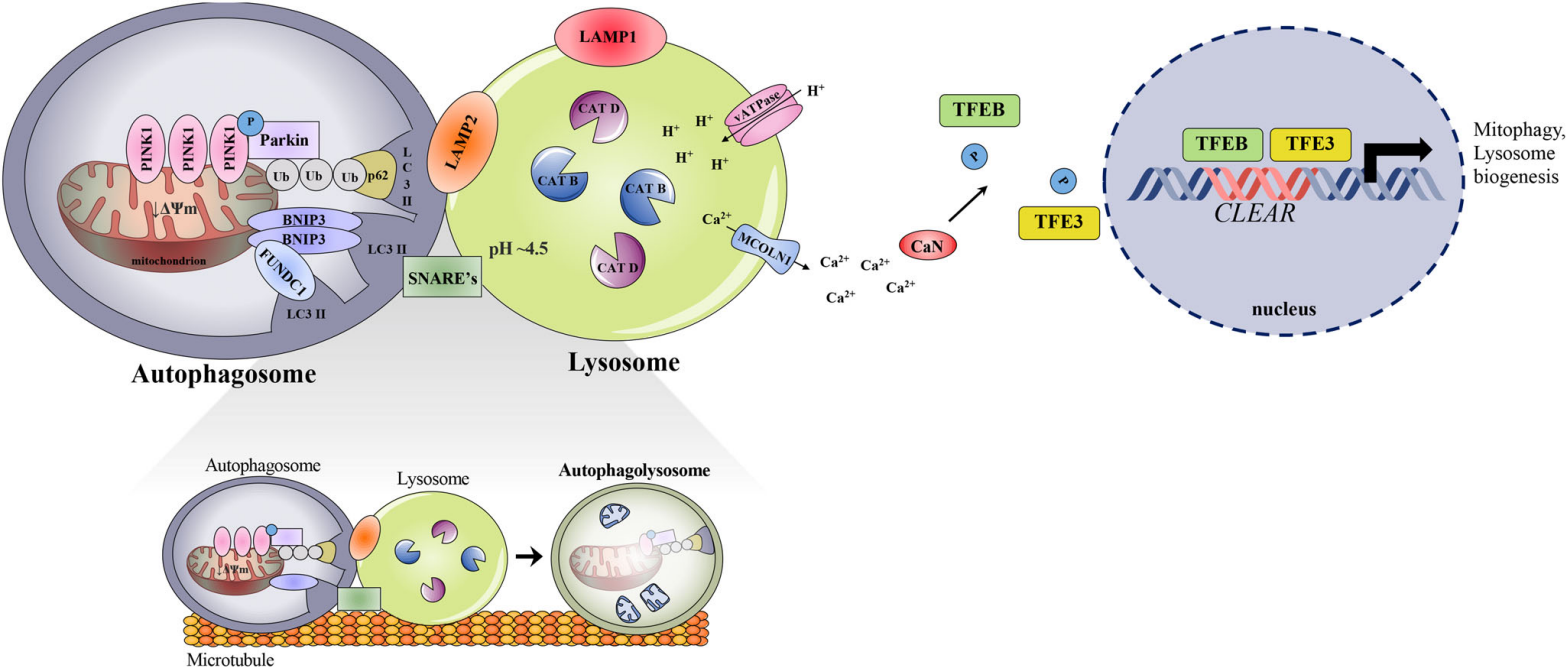
Regulation of mitophagy and lysosome biogenesis in skeletal muscle. Dephosphorylated transcriptional factor EB/E3 acts on the *Coordinated Lysosomal Expression and Regulation (CLEAR)* region of DNA to promote lysosomal biogenesis markers. Dysfunctional mitochondria characterized by a decreased mitochondrial membrane potential are engulfed by an autophagosome through the process of mitophagy. PINK1 will accumulate on the outer mitochondrial membrane owing to defective import and will recruit Parkin, an E3 ubiquitin ligase, which will tag outer mitochondrial membrane proteins. p62 is an adapter protein that binds to ubiquitins and LC3‐II, a marker of a mature autophagosome. BNIP3, when recruited to the mitochondria, will homodimerize and bind to LC3‐II. FUNDC1 also binds directly with LC3‐II, mediating mitophagy. Lysosomes are characterized by an acidic environment allowing for proteases and hydrolases, such as Cathepsin D and Cathepsin B, to degrade proteins. LAMP1 and LAMP2 are lysosomal membrane‐associated proteins. vATPase pumps hydrogen ions to maintain the acidic environment. MCOLN1 is a channel that releases calcium, which allows for the activation of Calcineurin (CaN), a phosphatase that can dephosphorylate TFEB and TFE3, decreasing their sequestration. Autophagosomes and lysosomes fuse along microtubules with the help of LAMP2 and SNAREs, resulting in an autophagolysosome, where the breakdown and recycling of mitochondria occurs.

Mitophagy is a selective form of autophagy whereby mitochondria are exclusively tagged for removal and degraded within an autophagolysosome, a structure composed of a fused autophagosome and lysosome. This is an important mechanism of MQC to prevent the accumulation of dysfunctional mitochondria, which can lead to a decline in overall respiratory function and skeletal muscle performance. Mitophagy pathways are essential for the initial tagging and selection of dysfunctional mitochondria (Figure 1).

### 2.1 Overview of mitophagy pathways

Muscle cells and other tissues possess two well‐characterized stress‐induced mitophagy pathways. The first is the PINK1/Parkin pathway, whereby in basal conditions PTEN‐induced kinase 1 (PINK1) is imported into the mitochondrion and cleaved via its mitochondrial targeting sequence (MTS) by mitochondrial processing peptide (MPP), and further cleaved by presenilin‐associated rhomboid‐like protease (PARL). When the mitochondrial membrane potential (ΔΨ_m_) is reduced, protein import is impaired, and PINK1 accumulates on the outer mitochondrial membrane (OMM). PINK1 will then recruit and activate Parkin by direct phosphorylation, allowing Parkin to act as an E3 ubiquitin ligase responsible for tagging proteins located on the OMM (Narendra et al., 2010). This ubiquitin chain will ultimately attract adapter proteins, such as p62, to bind to both the ubiquitin chain and microtubule‐associated protein 1 light chain 3 (LC3‐II) to initiate autophagosome formation, following maturation conversion from LC3‐I (Ju et al., 2010). The knockout of the *PRKN* gene in a mouse model leads to a reduction in mitophagy signalling in skeletal muscle, identifying the importance of this protein in mitophagy (Chen et al., 2018).

The second pathway is receptor‐mediated mitophagy through receptors located on the OMM. Various receptors, such as BNIP3 or FUNDC1, interact directly with LC3‐II to facilitate removal of mitochondria. BNIP3 homodimerizes in the mitochondrial outer membrane in order to recruit autophagosomes (Li et al., 2021; Sebastian et al., 2016). Mutation of the LC3–BNIP3 binding site reduces mitophagy flux, as seen by attenuation of the degradation of mature autophagosome marker LC3‐II (Hanna et al., 2012). Another mechanism of receptor‐mediated mitophagy is the FUNDC1 pathway. In homeostatic conditions, PARL is sequestered in the inner mitochondrial membrane to prohibitin 2 (PHB2) (Champsi et al., 2025); however, in stress‐induced conditions where there is a loss of ΔΨ_m_, PARL is released and cleaves PGAM5, leading to its translocation to the cytosol. PGAM5, a phosphatase, is then able to dephosphorylate FUNDC1, leading to its mitochondrial translocation. This allows for interaction with LC3‐II, initiating FUNDC1‐mediated mitophagy (Qi et al., 2025). FUNDC1 deletion in skeletal muscle leads to an impairment in autophagosome formation during stress‐induced conditions, emphasizing the importance of receptor‐mediated mitophagy (Fu et al., 2018). ULK1, a protein involved in the initiation of autophagosome formation, is required for the mitophagy response mediated through FUNDC1 as observed in ULK1 knockout skeletal muscle (Laker et al., 2017). In addition, the inner membrane phospholipid cardiolipin is also involved in mitophagy, and when it translocates to the outer mitochondrial membrane during mitochondrial damage it can bind directly to LC3, initiating selective organelle removal (Iriondo et al., 2022). Receptor‐mediated mitophagy within skeletal muscle is underexplored but seems to be crucial for mitophagy‐related adaptations.

### 2.2 Measuring mitophagy

The fusion of autophagosomes with lysosomes is the final step in mitophagy and it is mediated by SNAREs and lysosomal associated membrane protein 1/2 (LAMP1/2) isoforms found on the lysosomal membrane. Autophagosomes within LAMP1 and LAMP2 double‐deficient cells are unable to fuse with lysosomes, characterizing the importance of these proteins in the fusion process (Huynh et al., 2007). Autophagosomes travel along microtubules, where they are able to fuse and form autophagolysosomes (Lőrincz et al., 2020). Autophagy or mitophagy flux can be measured through the administration of microtubule inhibitors, such as colchicine, whereby microtubule growth is arrested, inhibiting fusion of the two organelles, leading to the accumulation of autophagy and mitophagy markers such as LC3‐II, p62 and Parkin (Chen et al., 2018; Leung et al., 2015). Bafilomycin A1 is another widely used inhibitor of autophagolysosome fusion via degradation of LC3‐II and the inhibition of lysosomal vATPase‐dependent acidification (Klionsky et al., 2008). Owing to the dynamic nature of the autophagy and mitophagy pathways, measuring flux using whole‐cell or tissue fractions is essential for the accurate assessment of these pathways. For example, the accumulation of autophagy‐related proteins, such as LC3‐II, p62 and Parkin, in isolated muscle mitochondria is indicative of mitophagy flux, given that these proteins are specifically involved in the tagging and degradation of mitochondria (Parousis et al., 2018; Stouth et al., 2024; Vainshtein et al., 2015; Wong et al., 2024). However, there are limitations to current methods of measuring mitophagy using inhibitors such as colchicine and bafilomycin A, because they can impact cellular functions, especially during long treatment times (Rahman et al., 2025). However, these effects can be minimized if they are used for relatively short periods of time, such as 3 days (Wong et al., 2024).

Measuring mitophagy markers without the ability to measure flux and their accumulation is a static representation of the pathway, and this is useful particularly in human studies for identifying the capacity of individual steps within mitophagy in various experimental models. In addition, several fluorescent reporter systems have also been developed as a method to measure mitophagy flux in vivo or in vitro. Mt‐Keima mice are a transgenic mouse model developed by Finkel and colleagues (Sun & Finkel et al., 2015). The fluorescent Keima protein is conjugated to the presequence of COX8, a mitochondrion‐specific protein located in the electron transport chain (ETC), and through dual‐excitation, single‐emission fluorescence, at physiological pH (∼7), such as in the cytosol, mitochondria will fluoresce green. When mitochondria are localized to the acidic environment of the lysosome (pH ∼4.5), they will fluoresce red, indicating that these mitochondria are undergoing mitophagy. *mito*‐QC mice represent an alternative transgenic mouse using a tandem‐fusion model (McWilliams & Ganley et al., 2016) that expresses green fluorescent protein (GFP) and mCherry fused to FIS1, an OMM protein. When mitochondria are found within the acidic environment of the lysosome, GFP will be quenched, and mCherry will fluoresce red. This was initially developed in vitro using a construct by the same group in 2013 (Allen & Ganley et al., 2013). MitoTimer is another model (Hernandez & Gottlieb et al., 2013) that can be used in vivo and in vitro for measuring mitophagy when co‐localized with lysosomal proteins. MitoTimer is targeted to mitochondria using a presequence from COX8, and it fluoresces green when newly synthesized, shifting to red when oxidized. Thus, the interpretation of data using MitoTimer is less specific to mitophagy per se, unless co‐localized with lysosomal markers (Laker et al., 2017). Thus, these multiple methods that exist for measuring mitophagy are advantageous for understanding mitophagic signalling in various conditions in skeletal muscle and other tissues.

### 2.3 Basal mitophagy

In basal conditions, where shifts in energy demand are not profound, mitophagy is a continuous process of removing dysfunctional mitochondria in an attempt to preserve a healthy mitochondrial reticulum. In skeletal muscle, basal mitophagy flux is negatively correlated with mitochondrial content (Mofarrahi et al., 2013; Sedraoui et al., 2024). Mitophagy flux indicated by mitochondrial and lysosomal co‐localization using a *mito*‐QC mouse model was significantly higher in the gastrocnemius and tibialis anterior, both mostly composed of type II fibres possessing reduced levels of mitochondria in comparison to the diaphragm, a higher oxidative muscle type (Sedraoui et al., 2024). Likewise, autophagic flux measured using colchicine was also observed to be higher in the fast‐twitch gastrocnemius and tibialis anterior muscles relative to the soleus (Mofarrahi et al., 2013).

PINK1 initiates mitophagy following stress‐induced mitochondrial depolarization. Loss of PINK1 in skeletal muscle leads to reduced basal mitophagy flux exclusively in highly oxidative tissue, indicating that PINK1‐induced mitophagy is dependent on oxidative fibre type (Singh et al., 2024). Observations made using the *Pink1* knockout *mito*‐QC mouse model (McWilliams et al., 2018) have indicated that mitophagy flux can occur independently of the PINK1 pathway in lower oxidative skeletal muscle. Thus, there seems to be a clear fibre‐type difference in the regulation of basal mitophagy, negatively correlated to mitochondrial content. Although the mechanisms underlying this fibre‐type difference are unclear, it seems that utilization of the PINK1–Parkin pathway in highly oxidative skeletal muscle might play a role in differentiating basal mitophagy in lower vs. higher oxidative fibre types. Peroxisome proliferator‐activated receptor‐γ coactivator‐1α (PGC1α) has also been observed to have a significant impact on basal mitophagy in skeletal muscle. In PGC1α knockout conditions, basal mitophagy was attenuated, alongside lower mitochondrial content and a myopathic phenotype (Vainshtein et al., 2015). Basal mitophagy has a ‘housekeeping’ purpose; however, using a stressor to understand the relationship between physiological conditions and mitophagy flux is equally important.

### 2.4 Stress‐induced mitophagy

Many different stimuli can induce mitophagy signalling pathways in skeletal muscle, all involving a change in energy demand based on physiological conditions. Acute exercise in vivo and stimulation‐induced contractile activity in myotubes are documented methods of inducing mitophagy signalling in a skeletal muscle setting. In C2C12 myotubes, bafilomycin A1 induced the accumulation of both LC3‐II and p62 in mitochondrial fractions after a 3 h bout of in vitro contractile activity (Oliveira et al., 2023). Acute exhaustive treadmill exercise in vivo has also been used in rodent models to investigate mitophagy flux. With the use of the MitoTimer mouse, co‐localization of fluorescent red mitochondria and LAMP1‐YFP (yellow fluorescent protein) was shown to occur in muscle after an acute bout of exercise (Laker et al., 2017). Experiments measuring mitophagy flux using colchicine demonstrated an accumulation of mitophagy‐related proteins, such as LC3‐II, p62 and Parkin, in mitochondrial isolations after an acute bout of exercise or in situ stimulation (Chen et al., 2018; Vainshtein et al., 2015; Wong et al., 2024). In the absence of a mitophagy blocker, exercise causes a reduction in the level of p62 (Triolo et al., 2022), which can be attributed to its accelerated degradation, because its function as an adapter protein undergoes mitophagy and subsequent degradation via the autophagolysosome.

Acute exercise induces metabolic changes that initiate the signalling towards the mitophagy pathway. These signals include transient increases in the activation of various kinases via phosphorylation. AMP‐activated protein kinase (AMPK) localizes to mitochondria after an acute bout of exercise in skeletal muscle, and the deletion of AMPK leads to an impairment in mitophagy, suggesting that its activation is necessary to facilitate mitophagy signalling pathways after an acute bout of exercise (Drake et al., 2021). Furthermore, models of disuse leading to changes in energy demand can be used to induce mitophagy. Unilateral sciatic denervation in rodent models upregulates autophagy flux particularly after short‐term (1–3 days) of denervation. At longer time points, this effect is reduced, suggesting that early changes in mitophagy are important for the maintenance of mitochondrial health (Triolo et al., 2022). Mitophagy can also be induced by conditions of energy deprivation, such as those imposed by fasting (Stouth et al., 2024). Indeed, stress‐induced mitophagy appears to be evident in physiological conditions that perturb energy balance, both expenditure and deprivation, to preserve mitochondrial homeostasis.

## 3 ROLE OF LYSOSOMES

Lysosomes are membrane‐bound organelles containing a variety of digestive enzymes functional at an acidic pH (∼4.5) that are responsible for the breakdown of cargo and debris within the cell (Bainton, 1981). Lysosomes are the terminal step in autophagy and mitophagy, ultimately responsible for the degradation of dysfunctional mitochondria. The initial steps in mitophagy have been well characterized in skeletal muscle; however, the steps involving lysosomal degradation and recycling have been under‐studied in muscle particularly in various physiological conditions, such as exercise, age or disuse (Moradi et al., 2024).

### 3.1 Lysosome structure and function

The formation of lysosomes occurs through lysosomal biogenesis or via various fusion or maturation processes from endosomes. Endosomes are vesicles that contain specific proteins, such as the mannose‐6‐phosphate receptor (M6PR) and GTPase Ras‐related protein 5 (Rab5), that are involved in the trafficking of endosomal and lysosomal enzyme. These proteins are not found in late endosomes or lysosomes, thus allowing for the biochemical differentiation between endosomes and lysosomes. Rab5 is replaced by Rab7 in late endosomes and lysosomes, where in its active state it is recruited to the membrane to assist in membrane trafficking or fusion, characterizing another marker of lysosomal differentiation (Luzio et al., 2007). In C2C12 myotubes, the co‐localization of Rab7 and lysosomal associated membrane protein 1 (LAMP1) was observed, indicating these proteins to be lysosomal membrane markers (Irazoki et al., 2022). Lysosomal associated membrane proteins 1 and 2 (LAMP1 and LAMP2) are specific to lysosomes and are involved in the housing and trafficking of various lysosomal hydrolases. LAMP1 and LAMP2 are heavily glycosylated, making their molecular weights ∼100 kDa, whereas their unglycosylated forms are ∼45 kDa (Eskelinen, 2006). The glycosylation status of these proteins is an important characteristic of lysosomal integrity, reducing their potential degradation within the proteolytic environment of the lysosome (Cawley et al., 2020). When LAMP1 and LAMP2 protein levels are reduced via knockdown technology, the differentiation of C2C12 myoblasts to myotubes is impaired, and this appears to be attributable predominantly to the absence of LAMP2 (Sakane et al., 2018). The LAMP proteins have been shown to be involved in autophagosome and lysosome fusion; however, LAMP1 is dispensable from this mechanism if LAMP2 is present, indicating that LAMP2 has a more dominant role for autophagolysosome fusion (Chaudhry et al., 2022). Lysosomal integral membrane protein 2 (LIMP2) is another highly glycosylated protein found within the lysosomal membrane. The glycosylation status of LIMP2 is suggested to be characteristic of an intact and functional lysosome (Gonzalez et al., 2014). This is attributable primarily to its interaction with glucocerebrosidase (GCase), a lysosomal hydrolase, which is dependent on LIMP2 to be delivered to the lysosome (Boer et al., 2020).

Lysosomes have an acidic environment with a pH of ∼4.5, which is a necessary characteristic for their function. This acidity is important for the function of lysosomal enzymes and hydrolases responsible for the degradation of cargo (Fernández et al., 2023). Vacuolar ATPase (vATPase) uses ATP to pump protons (H^+^) into the lysosomal lumen to regulate lysosomal acidification. Inhibition or inactivation of vATPase causes lysosomal dysfunction owing to the inability of hydrolases to function. Several ion channels are also involved in regulating the acidic balance in lysosomes (Mindell, 2012). Transmembrane protein 175 (TMEM175) functions to efflux protons and has been shown to interact directly with LAMP1 and LAMP2, which inhibit the activity of TMEM175 in order to maintain an acidic environment (Zhang et al., 2023). Mucolipin 1 (MCOLN1, also called transient receptor potential mucolipin 1, TRPML1) is a channel involved in the release of cations, such as calcium (Ca^2+^), further regulating the lysosomal lumen (Qi et al., 2024). Two‐pore channel subtype 2 (TPC2) is also involved in mediating Ca^2+^ release. These various channels are found on both endosomes and lysosomes (Chen et al., 2022).

Hydrolase activity defines the primary function of lysosomes to break down various debris or cargo, such as dysfunctional mitochondria, during the process of mitophagy to prevent their cellular accumulation. Among the hydrolases that exist, the family of cathepsin enzymes are the most abundant proteases found in lysosomes (Yadati et al., 2020). The cathepsin isoforms L, B and D are most abundant and are initially synthesized as pre‐pro‐enzymes and transported to the endoplasmic reticulum. The pre‐peptide is cleaved, and a pro‐enzyme is formed, which is typically referred to as the immature form of a cathepsin and is enzymatically inactive. Cathepsins have mannose‐6‐phosphate (M6P) residues, which allow them to bind to M6PR on endosomes, where the mild acidic pH (∼6.0) removes the M6P. After the conversion of an endosome to a lysosome, the cathepsins undergo proteolytic processing into their active mature forms either by auto‐activation, whereby the acidic pH enables cleavage of the pro‐peptide by the catalytic site, or by trans‐activation, which is proteolytic activity with the help of other proteases. The mature forms of certain cathepsins, such as cathepsin B, are able to function only in acidic environments, thus any enzyme released from the interior of the lysosome will impede their proteolytic activity (Yadati et al., 2020). Lysosomal proteolytic activity determined via lumen acidity is an important aspect of mitophagy because it mediates the degradation of dysfunctional mitochondria.

### 3.2 Lysosomal biogenesis

Lysosomal biogenesis is the addition of newly synthesized lysosomes to the current pool in order to maintain lysosomal health and cellular degradation capacity. This process is transcriptionally controlled by the microphthalmia/transcription factor E (MiTF/TFE) family, which is composed of transcription factor EB (TFEB), transcription factor TFE3, microphthalmia‐associated transcription factor (MITF) and transcription factor EC (TFEC). These transcription factors can homo‐ or hetero‐dimerize and bind to the Coordinated Lysosomal Expression and Regulation (CLEAR) motif involved in the transcription of lysosomal and autophagy markers (Agostini et al., 2022). In C2C12 myotubes, the induction of mitophagy is impaired, and lysosomal markers are downregulated with the loss of TFEB and TFE3, elucidating the importance of these transcription factors in mediating both mitochondrial and lysosomal homeostasis (Oliveira et al., 2023). The activity of these transcription factors in the nucleus is controlled, in part, by the mammalian target of rapamycin complex 1 (mTORC1), a kinase that phosphorylates TFEB and TFE3 at serine^211^ and serine^321^, respectively. When phosphorylated at these sites, TFEB and TFE3 are bound to 14‐3‐3 proteins and are cytosolically sequestered, preventing nuclear translocation. TFEB and TFE3 can be dephosphorylated at these serine residues by calcineurin to promote their nuclear translocation (Martina et al., 2016). In contrast, AMPK is an enzyme that phosphorylates both TFEB and TFE3 on serine^466^, serine^467^ and serine^469^ which, in these cases, allows for their nuclear translocation (Paquette et al., 2021). The balance in phosphorylation of these serine residues will determine TFEB or TFE3 nuclear translocation or cytosolic sequestration based on physiological stimuli.

Acute exercise has been shown to induce lysosomal biogenesis via TFEB nuclear translocation (Triolo et al., 2022). Exercise promotes this via the activation of various signalling pathways. For example, acute exercise increases ATP turnover, leading to increased levels of AMP and ADP and decreased ATP levels (Richter et al., 2009). This activates AMPK, which phosphorylates mTORC1, suppressing its activity, leading to the cytosolic release of TFEB and TFE3 and promoting their nuclear translocation (Agarwal et al., 2015). In addition, the activation of MCOLN1 has been shown to lead to lysosomal calcium release, which can activate calcineurin to dephosphorylate TFEB and promote its nuclear localization (Medina et al., 2015). Therefore, exercise is a potent regulator of the lysosomal biogenesis pathway, essential for the synthesis of mitophagy and lysosomal markers.

### 3.3 Measuring lysosomal function

The function of lysosomes is important for regulating mitophagy, because the breakdown, degradation and recycling of dysfunctional mitochondria can occur only if lysosomal enzymes are active and functional following their fusion with an autophagosome. Measuring lysosomal function can be done in various ways. For example, Lysotracker is a cell‐permeable fluorescent dye that stains acidic compartments within a cell, such as lysosomes and late endosomes. This dye can be used to label and visualize intact lysosomes using confocal microscopy (Huang et al., 2024). Alternatively, measurement of Ca^2+^ release from TRPML/MCOLN1 has been used as an indicator of lysosomal function with respect to the ability of the channel to induce downstream Ca^2+^ signalling (Bhattacharjee et al., 2024). In addition, the use of enzymatic assays to measure lysosomal hydrolase activity allows for an indication of how well the hydrolase is able to cleave a given peptide. Specifically, Cathepsin B/L assays have been used to indicate the ability of lysosomes to function proteolytically (Gumpper et al., 2017). These assays would reveal organelle‐specific functionality if done in isolated lysosomal fractions, akin to the measurement of oxygen consumption rate in isolated mitochondrial fractions. Recently, a new method for the isolation of relatively pure lysosomes from skeletal muscle has been described (Mahendran et al., 2025). The combination of multiple enzymatic assays using isolated lysosomes will be useful to create a complete picture of lysosomal function in muscle in health, adaptation, exercise and disease and is important for understanding the role that lysosomes have in the terminal step of mitochondrial degradation.

## 4 ADAPTATION

**FIGURE 2:**
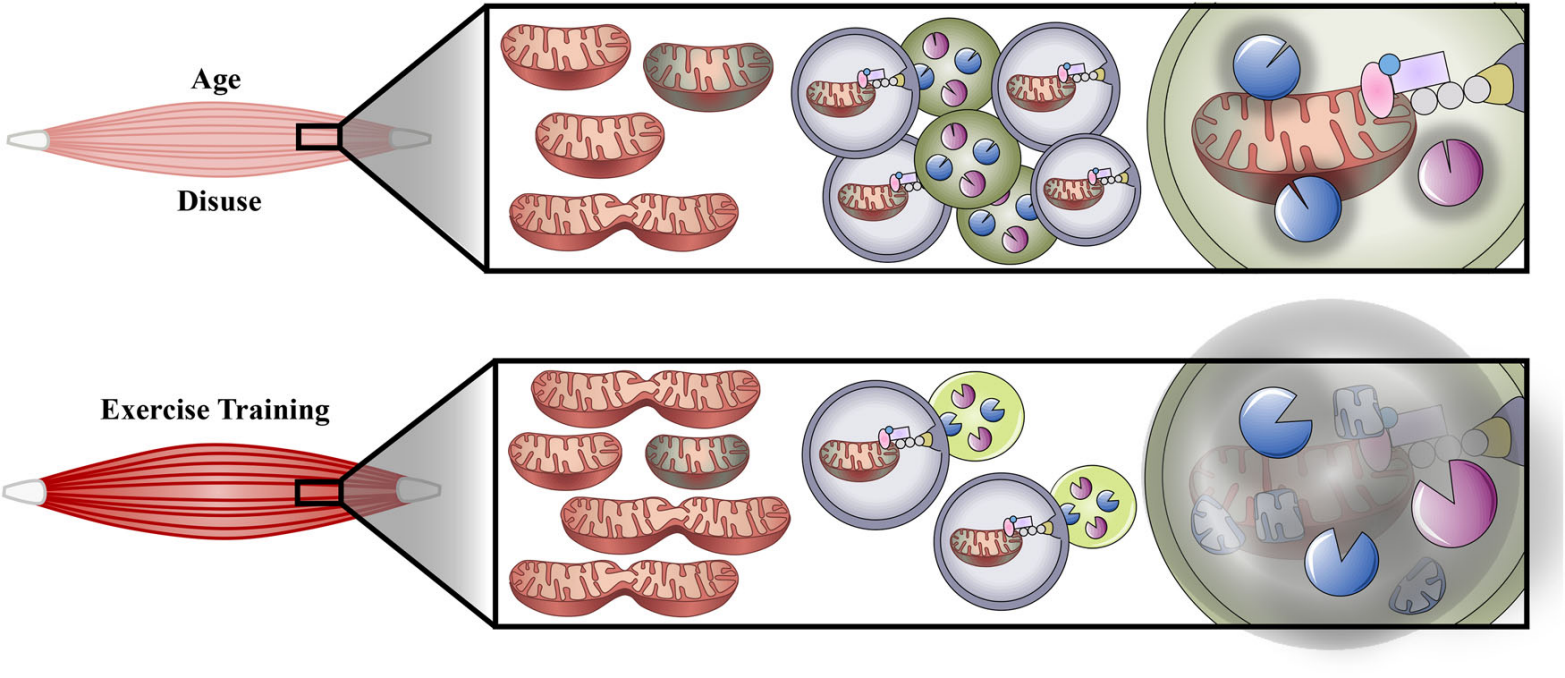
Skeletal muscle, mitochondrial, mitophagy and lysosomal adaptations with age, exercise and disuse. Skeletal muscle is highly adaptable to various physiological changes, largely attributed to the plasticity of the mitochondrial reticulum. Ageing and disuse lead to a loss of muscle mass accompanied by increases in mitophagy markers and dysfunctional lysosomes with poorly functioning proteolytic activity, impacting and attenuating turnover. Exercise training increases mitochondrial content, basal mitophagy levels and lysosomal proteolytic activity, increasing turnover, thus improving the mitochondrial reticulum.

Mitochondria are highly adaptable to various physiological conditions. Periods of chronic muscle use and/or disuse, in addition to the naturally occurring ageing process, have all been shown to exert adaptive effects on the mitochondrial reticulum (Hood et al., 2019) (Figure 2). Adaptations of mitochondria are a result of changes in organelle ‘turnover’, the balance between mitochondrial biogenesis (i.e., synthesis) and mitophagy. Both these processes are highly adaptive, and they regulate the metabolic health status of skeletal muscle.

### 4.1 Age‐related adaptations

Whether changes in mitochondrial content and function occur with age or not is a highly controversial topic, with conclusions found in the literature that are dependent on the age in question, in addition to the level of physical activity of the subjects. For example, human studies may report a decline in mitochondrial content or function (Conley et al., 2000; Crane et al., 2010) or they may not (Distefano et al., 2016; Gouspillou et al., 2014; Ringholm et al., 2023 ), depending on how active the subjects are (Distefano et al., 2018). Nonetheless, primary mitochondrial defects attributable to ageing alone in human muscle have been compiled and thoroughly reviewed in recent publications (Carter et al., 2015; Hood et al., 2016). In rodent models of ageing, a naturally progressive change in mitochondrial bioenergetics has been documented (Chabi et al,. 2008; Chen et al., 2018), which cannot be completely reversed with contractile activity (Ljubicic et al., 2009). In addition, contractile activity‐induced signalling towards mitochondrial biogenesis is impaired (Ljubicic et al., 2009), and cytosolic mitochondrial DNA levels are elevated (Khemraj et al., 2025) in ageing muscle, suggestive of mitochondrial defects. Mitochondrial biogenesis mediated by PGC‐1α is blunted in aged skeletal muscle (Carter et al., 2018), leading to a diminished drive for the synthesis of new mitochondria. These data indicate a cellular requirement for accelerated mitophagy to remove dysfunctional organelles. There is evidence for accelerated mitophagy flux in ageing muscle to accommodate this need (Carter et al., 2018; Chen et al., 2018) in studies using colchicine inhibition of the microtubule pathway method to measure flux. Other studies using a *mito*‐QC model animal model (Jiménez‐Loygorri et al., 2024) or using whole‐tissue fractions (Baninameh et al., 2024) suggest that there are no changes in flux in aged gastrocnemius muscle. Regardless of pathway flux, the process itself appears to be limited by lysosomal function, the terminal degradative organelle of mitophagy. Evidence of lysosomal dysfunction in aged muscle is observed via electron microscopy with the presence of lipofuscin, a marker of accumulated non‐degraded lysosomal content (O'Leary et al., 2013) and reductions in cathepsin activity (Mahendran et al., 2025). This is accompanied by elevated levels of lysosomal markers, in apparent compensation for organelle dysfunction (Carter et al., 2018; Triolo et al., 2022).

TFEB, a regulator of both mitophagy and lysosomal gene expression, is increased in aged skeletal muscle and is more abundant in nuclear fractions relative to young, suggesting that this transcription factor is a primary driver of the increase in lysosomes with age (Carter et al., 2018; Triolo et al., 2022). Proteins involved in mitophagy, such as PINK1, Parkin, p62 and LC3‐II, are degraded in the lysosome; however, if degradation is not occurring normally these will accumulate, as observed when mitophagy is blocked in studies that use colchicine (Wong et al., 2024). Lysosomal impairments with age have also been documented with respect to proteolytic activity, acidification and quality control, including lysosomal biogenesis and lysophagy, the degradation of dysfunctional lysosomes (Tan et al., 2023). In aged skeletal muscle, diminished proteolytic activity has been observed via reduced cathepsin B and L activities relative to young muscle, based on the ability of these enzymes to cleave a peptide substrate (Fernando et al., 2020; Mahendran et al., 2025). Lysosomal proteases require an acidic environment in order to mature and function properly. Cathepsins are able to cleave into their mature, proteolytically active form only within an environment with sufficient acidification (Crombie et al., 2023). Acidity levels within the lysosome are maintained through vacuolar‐type ATPase (vATPase), and its expression is correlated with increased longevity (Sun et al., 2020). Although vATPase is upregulated in aged muscle, its activity has been shown to be altered (Colacurcio et al., 2016). These studies suggest that there is an impairment in lysosomal acidification leading to reduced proteolytic activity within aged skeletal muscle. Recently, direct assessments of organelle function using isolated, purified lysosomes have also revealed reduced proteolytic capacity in aged muscle (Mahendran et al., 2025). The degree to which this occurs in either human or rodent muscle will also be a determinant of whether an accumulation of dysfunctional mitochondria will be evident. Thus, it is clear that an understanding of mitophagy and lysosomal changes with age is important to elucidate what might be driving changes in the metabolic health of aged skeletal muscle.

### 4.2 Exercise adaptations

Endurance exercise increases mitochondrial content and function through changes in mitochondrial biogenesis and turnover via mitophagy (Hood et al., 2019). Exercise increases the expression of transcription factors responsible for mitophagy and lysosomal biogenesis, such as TFEB and TFE3, in addition to their translocation to the nucleus (Triolo et al., 2022). Downstream markers, such as Parkin, Beclin1, lysosomal membrane and cathepsin markers, have been shown to be elevated in trained muscle (Balan et al., 2019; Chen et al., 2018; Parousis et al., 2018; Wong et al., 2024). In a human study evaluating sedentary versus endurance‐trained individuals, analyses indicated increases in mitophagy markers, such as PINK1 and Parkin, correlating with a higher mitochondrial content found in trained individuals (Tarpey et al., 2017). In conditions where TFEB and TFE3 are reduced, exercise‐mediated adaptations do not occur, further elucidating the importance of the capacity for exercise‐mediated lysosomal biogenesis (Oliveira et al., 2023; Wong et al., 2024).

Acute exercise increases mitophagy flux benefitting the removal of dysfunctional mitochondria. This is displayed through an upregulation in mitophagy markers localized to mitochondria, such as Parkin, p62 and LC3‐II (Chen et al., 2018; Wong et al., 2024). In chronic exercise‐trained skeletal muscle, there are elevated levels of mitochondrial Parkin basally, which acute exercise does not increase further (Chen et al., 2018). This suggests that chronic endurance exercise training increases mitophagic capacity in basal conditions, leading to no requirement for further increases in Parkin translocation to the mitochondria, potentially owing to improved mitochondrial quality. However, no changes in mitophagy markers in human skeletal muscle were observed in resistance exercise training models (Mesquita et al., 2020). This suggests that not all exercise modalities have the same impact on mitophagy flux.

Using a model of chronic contractile activity, an increase in lysosomal markers occurs in as little as 3 days of chronic contractile activity, preceding mitochondrial adaptations (Carter et al., 2018; Kim et al., 2017; Parousis et al., 2018). As noted above, lysosomal function is defined largely by its proteolytic activity, which is determined by sufficient lysosomal acidification via vATPase. Chronic exercise has been shown to increase vATPase protein expression, thereby potentially improving the acidity of lysosomes (Wong et al., 2024). Although lysosomal proteolytic adaptations have not yet been elucidated with respect to chronic exercise, an acute bout of exercise leads to an upregulation in cathepsin D activity (Dohm et al., 1980). This suggests that over time with chronic exercise, proteases might adapt to have increased activity owing to improvements in lysosomal acidification mediated by vATPase.

### 4.3 Disuse adaptations

Chronic states of disuse, such as immobilization or denervation, lead to muscle atrophy. This is determined by an imbalance in protein turnover, favouring degradation rather than synthesis. The decrements in mitochondrial content and function that accompany muscle disuse contribute meaningfully to the atrophy process via the activation of apoptotic signalling and proteolytic pathways and via energy deficits and downstream signalling (Powers et al., 2012). Thus, there is a requirement for the removal of dysfunctional mitochondria via mitophagy, and mitophagy‐related proteins, such as Beclin1, PINK1, Parkin, LC3‐II and p62, are all elevated in conditions of chronic disuse (Memme et al., 2022; Oliveira et al., 2024; Rosa‐Caldwell et al., 2020). Lysosome‐related proteins, such as TFEB, LAMP1, LAMP2 and cathepsin D, are also upregulated in conditions of disuse (Triolo et al., 2022). Interestingly, during denervation‐induced muscle disuse the increase in mitophagy flux is early and transient and is followed by increases in lysosomal markers (Triolo et al., 2022). These data suggest that lysosomes play an important role in mediating mitophagy during disuse adaptations in an attempt to remove dysfunctional mitochondria. Studies of disuse in the presence of inhibited mitophagy have noted even further reductions in mitochondrial respiration, increased levels of reactive oxygen species, and muscle atrophy. This illustrates the protective effect that the mitophagy pathway exerts in maintaining muscle health (Rahman et al., 2025).

In human skeletal muscle subject to immobilization, BNIP3 and LC3‐II co‐localization is indicative of mitophagy and is observed to occur (Wang et al., 2023); however, others have not noted changes in mitophagy markers, such as Parkin, in human models of bedrest (Noone et al., 2025). Given that human studies must rely on static measurements of mitophagy markers rather than flux measurements, it is important to use markers that are strongly correlated with flux measures as determined in cell or animal models. Recent work has highlighted that the use of a combination of both p62 and LC3‐II in whole‐muscle extracts or in isolated mitochondrial fractions can closely predict flux measures in conditions of muscle disuse (Memme et al., 2022), an observation that could be useful in human studies of autophagy and mitophagy.

## 5 CONCLUSION

Mitophagy is an important mechanism responsible for the degradation of dysfunctional mitochondria. This process involves the selective tagging, engulfment and trafficking of dysfunctional organelles towards the lysosome, in which the terminal steps of degradation take place via specialized proteolytic activity. All stages of mitophagy are important processes for mitochondrial quality control and are highly adaptable to changes in physiological conditions. Recent research has begun to delve into the important yet relatively ignored role of the lysosome in mediating both the degradation of cargo and in initiating multiple retrograde signalling pathways towards the nucleus. Ageing, exercise training and muscle disuse all induce adaptations in mitophagy and lysosomal functions to meet the extensive changes in skeletal muscle mitochondrial energy demands. Research in these areas is still emerging, and it will be exciting to unravel the mechanisms involved in these adaptations with implications for the sustenance of muscle health in ageing and disease.

## AUTHOR CONTRIBUTIONS

Anastasiya Kuznyetsova and David A. Hood were responsible for the conception and design of the work, drafting the work and revising it critically, have approved the final version of the manuscript and agree to be accountable for all aspects of the work in ensuring that questions related to the accuracy or integrity of any part of the work are appropriately investigated and resolved. All persons designated as authors qualify for authorship, and all those who qualify for authorship are listed.

## CONFLICT OF INTEREST

None declared.
